# Supplementary material for: Shared Treg dysregulation underlies psoriasis vulgaris and atopic dermatitis through the PHF19-PRC2 epigenetic axis
Source: Front Immunol. 2026 Aug 20;17:1932788. doi: 10.3389/fimmu.2026.1932788 (PMC13538097; doi:10.3389/fimmu.2026.1932788)
Supplement: Supplementary file 3 [file DataSheet3.zip › Additional files/Additional file 8/33.CDKN2D.leaveoneout.pdf]

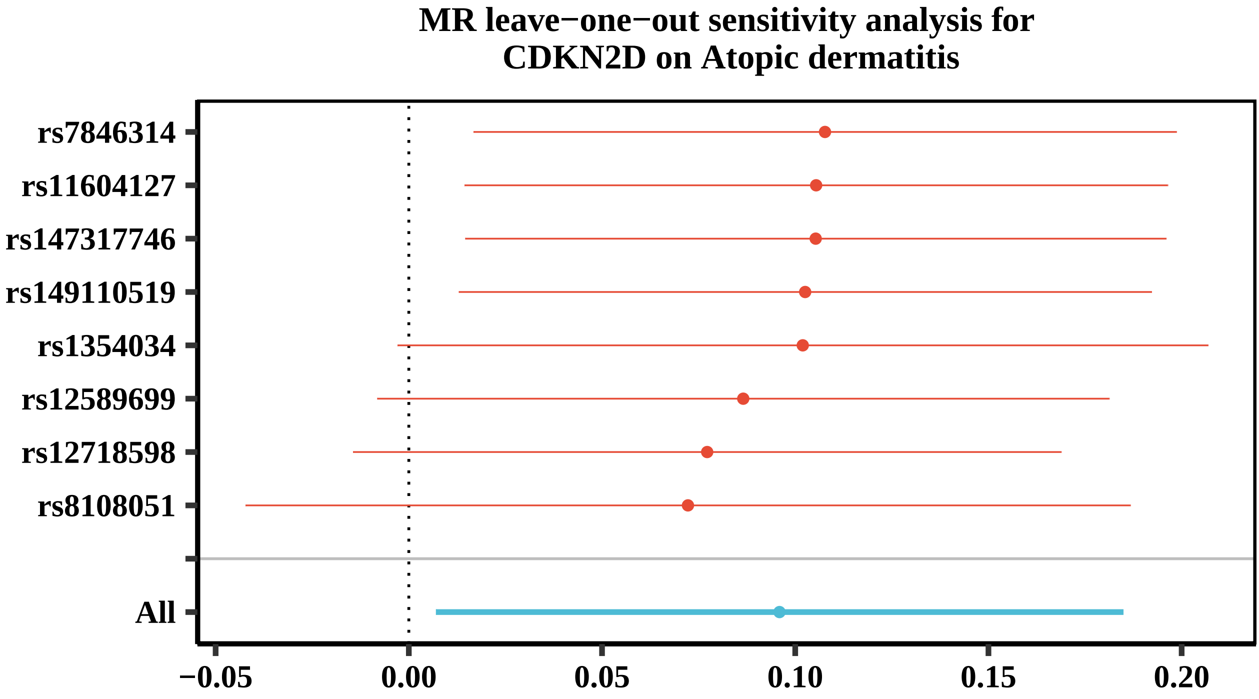
MR leave−one−out sensitivity analysis for
CDKN2D on Atopic dermatitis
rs7846314
rs11604127
rs147317746
rs149110519
rs1354034
rs12589699
rs12718598
rs8108051
All
−0.05
0.00
0.05
0.10
0.15
0.20
